# Peripheral Augmentation Index is Associated With the Ambulatory Arterial Stiffness Index in Patients With Hypertension

**DOI:** 10.4021/cr92w

**Published:** 2011-09-20

**Authors:** Kevin S. Heffernan, Eshan A. Patvardhan, Richard H. Karas, Jeffrey T. Kuvin

**Affiliations:** a Human Performance Laboratory, Department of Exercise Science, Syracuse University, Syracuse NY, USA; b Division of Cardiology and the Molecular Cardiology Research Institute, Tufts Medical Center, Boston MA, USA

**Keywords:** Augmentation index, Ambulatory arterial stiffness index, Blood pressure, Pulse wave amplitude

## Abstract

**Background:**

Vascular dysfunction is highly prevalent if not ubiquitous in patients with hypertension. We compared two different measures of vascular function obtained from digital volume waveforms with measures of ventricular-vascular load derived from 24-hour blood pressure (BP) recordings in patients with hypertension.

**Methods:**

Digital pulsatile volume waveforms were captured via plethysmography (peripheral arterial tone, PAT) and used to derive augmentation index (a measure of ventricular-vascular coupling) and the pulse wave amplitude-reactive hyperemia index (a measure of microvascular reactivity). Ambulatory arterial stiffness index (AASI) and the BP variability ratio (BPVR) were derived from 24-hour ambulatory BP recordings.

**Results:**

There was a positive association between PAT-AIx and AASI (r = 0.52, P < 0.05). There was also a positive association between PAT-AIx and BPVR (r = 0.37, P < 0.05). PAT-AIx was not associated with PWA-RHI (r = -0.14, P > 0.05). PWA-RHI was not associated with AASI or BPVR (P > 0.05).

**Conclusions:**

PAT-AIx is associated with ambulatory measures of vascular function and may offer clinical insight into vascular burden and cardiovascular disease risk in patients with hypertension independent of information obtained from PWA-RHI.

## Introduction

The digital volume waveform provides clinically useful information on peripheral vascular function and cardiovascular disease (CVD) risk. Peripheral arterial tone (PAT) makes use of pneumatic finger cuffs to assess volume change of the whole of the digit enclosed within the device (EndoPAT 2000, Itamar Medical Ltd), non-invasively generating a pulse volume waveform. Change in the amplitude of this volume waveform following a regional ischemic stimulus (i.e. pulse wave amplitude reactive hyperemia index; PWA-RHI) is a measure of resistance vessel reactivity that is associated with peripheral and coronary vascular function and is also a predictor of CV risk and future CV events [1-3].

A second measure easily obtained from the digital volume waveform using PAT is the augmentation index (AIx). As a measure of systemic vascular function and vascular-ventricular coupling, the AIx is a useful therapeutic target and secondary endpoint associated with numerous cardiovascular morbidities and mortality [4-6]. Although typically derived from the contour of central and/or peripheral arterial pressure waveforms, the AIx can also be derived from digital pulse volume waveforms using PAT. While the clinical and physiologic correlates of PWA-RHI have been well examined [7-9], less is known regarding the systemic vascular correlates of AIx derived from digital volume waveforms using PAT.

The ambulatory arterial stiffness index (AASI) is an integrated measure of systemic vascular function derived from 24-hour blood pressure recordings. This measure has been proposed to reflect dynamic arterial stiffening, left ventricular function/cardiac contractility, vascular resistance, wave reflection and overall vascular-ventricular coupling [10-13]. Although use of AASI as a measure of arterial stiffness has been challenged [14], this measure of vascular function is associated with target organ damage [15-18] and is an independent predictor of adverse CV events (i.e. stroke and CV mortality) [19-22]. The purpose of this study was to compare and contrast the association of vascular measures derived from PAT (AIx versus PWA-RHI) as they relate to the ambulatory arterial stiffness index (AASI) in patients with hypertension.

## Methods

Twenty four individuals with a history of hypertension (defined as SBP > 140 mmHg or DBP > 90 mmHg or being on an antihypertensive medication) recruited from the Tufts Medical Center preventive cardiology clinic, participated in this study. Exclusion criteria included ongoing myocardial ischemia, moderate to severe heart failure (NYHA class III-IV, LV ejection fraction < 35%), severe valvular disease, peripheral arterial disease (ankle-brachial index < 0.9), LDL-cholesterol > 100 mg/dL, unstable cardiac symptoms, renal insufficiency (serum creatinine > 2 mg/dL), active liver disease, chronic obstructive pulmonary disease, bronchial asthma, uncontrolled hypertension defined as baseline BP > 190/100 mmHg, Raynaud’s disease and/or finger deformities. This study was approved by the Institutional Review Board at Tufts Medical Center. Written informed consent was obtained from all the study participants prior to obtaining vascular measures.

The presence or absence of the following cardiovascular risk factors was assessed in each subject: gender, hyperlipidemia (total serum cholesterol > 240 mg/dL or taking lipid lowering medication), coronary artery disease (defined as the presence of ischemia or infarction on single-photon emission computed tomographic nuclear myocardial perfusion imaging or > 50% stenosis of an epicardial coronary artery by angiography), family history of CAD (having first or second degree relatives with CAD), and smoking status (having smoked at least five times per day within the last month).

### Finger pulse wave amplitude

Beat-by-beat pulse wave amplitude was captured in all patients using finger arterial tonometry (EndoPAT, Itamar Medical Ltd., Israel) as previously described in detail [1]. With patients in the supine position, plethysmographic finger cuffs were placed on the index fingers of both hands. A computerized algorithm automatically identified peak volume and inflection points using a 4 th order derivative as previously described by Kelly et al [23] and Takazawa et al [24]. Augmentation index was calculated from PWA waveforms as the ratio of the difference between the early and late systolic peaks of the waveform relative to the early peak expressed as a percentage (P_2_ – P_1_/P_1_ * 100).

The pulse wave amplitude-reactive hyperemia index (PWA-RHI) was calculated as the ratio of the average PWA over a 1-minute epoch starting after 5-minute of ischemia induced by brachial cuff inflation to a supra-systolic BP, divided by the average PWA of a 3.5 minutes baseline epoch. The PWA obtained from the finger of the non-occluded arm was also measured continuously and served as a control signal. Final values were normalized to the contra-lateral hand to account for any drift in the magnitude of the signal due to systemic factors. This was done automatically using customized computer software.

### Ambulatory blood pressure monitoring

Ambulatory BP was monitored in 24 patients with a commercially available device (North Atlantic Medical, Tolman Clinical Laboratory Cardiac Services, MA) once every 20-minute during the day (between 6 am and 10 pm) and once every 60-minute during the night (between 10 pm and 6 am). Ambulatory arterial stiffness index (AASI) was computed as the slope of the regression line (not forced through the origin) of systolic versus diastolic BP [21, 25]. AASI was taken as 1-regression slope. Systolic and diastolic blood pressure variability was defined as the standard deviation of ambulatory systolic and diastolic values, respectively. The blood pressure variability ratio (BPVR) was defined as SBP variability/DBP variability [26, 27]. Nocturnal dipping was defined as a reduction in average systolic and/or diastolic BP at night > 10% compared with average awake values and was treated as a discrete variable.

### Statistical analysis

Normality of distribution was assessed using Kolmogorov-Smirnof and Shapiro-Wilk tests. Group comparisons were made using analysis of variance with Tukey post hoc testing where appropriate. Chi-square tests were used to compare categorical variables. If significant group differences in potential confounders existed, analysis of covariance was used to statistically remove the influence of these parameters of outcome variables of interest. Pearson’s correlation coefficients were used to assess relationships between variables of interest. All data are reported as means ± SEM. A priori significance was set at P < 0.05.

## Results

**Figure 1:**
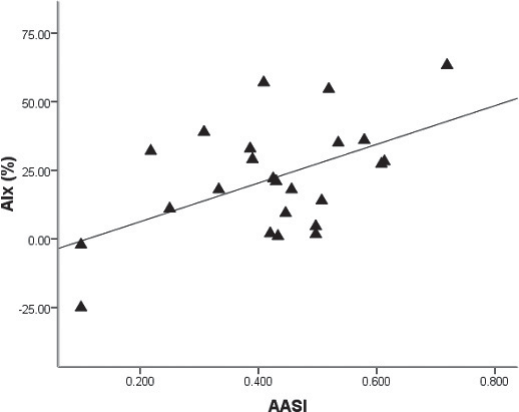
Association between peripheral arterial tonometery (PAT) derived augmentation index (AIx) and ambulatory arterial stiffness index (AASI).

Patient descriptive characteristics are presented in Table 1. There was a positive association between PAT-AIx and AASI (Fig. 1, r = 0.52, P < 0.05). According to partial correlation, this association remained after adjusting for potential confounders: age, body mass index, heart rate, and mean arterial pressure (r = 0.56, P < 0.05). There was a positive association between PAT-AIx and SBPV/DBPV ratio (r = 0.37, P < 0.05). PWA-RHI was not associated with PAT-AIx (r = -0.14, P > 0.05), AASI (r = 0.09, P > 0.05) or SBPV/DBPV ratio (r = 0.15, P > 0.05).

**Table 1:** Patient Descriptive Characteristics

Variable	All n = 24	Nocturnal Dippers n = 11	Nocturnal Non-Dippers n = 13
Age, years	56 ± 2	54 ± 4	58 ± 3
Male, %	71	64	77
BMI, kg/m^2^	28 ± 1	29 ± 2	27 ± 2
SBP, mmHg	140 ± 2	138 ± 2	141 ± 3
DBP, mmHg	80 ± 2	80 ± 2	80 ± 2
HR, bpm	75 ± 2	71 ± 3	77 ± 4
Total cholesterol, mg/dL	164 ± 8	177 ± 14	154 ± 8
LDL cholesterol, mg/dL	90 ± 7	95 ± 14	85 ± 7
HDL cholesterol, mg/dL	46 ± 3	49 ± 5	44 ± 5
Triglycerides, mg/dL	124 ± 21	135 ± 38	115 ± 21
CAD, %	46	45	46
Current smoker, %	33	18	46
Family history CVD, %	33	18	46
Medications, %			
Aspirin	75	82	69
Statin	71	73	69
Anti-hypertensive agent	100	100	100
AASI, au	0.42 ± 0.03	0.39 ± 0.04	0.45 ± 0.05
BPRV, %	1.30 ± 0.05	1.30 ± 0.06	1.30 ± 0.08
PWA-RHI	1.94 ± 0.2	2.08 ± 0.3	1.83 ± 0.3

**Figure 2:**
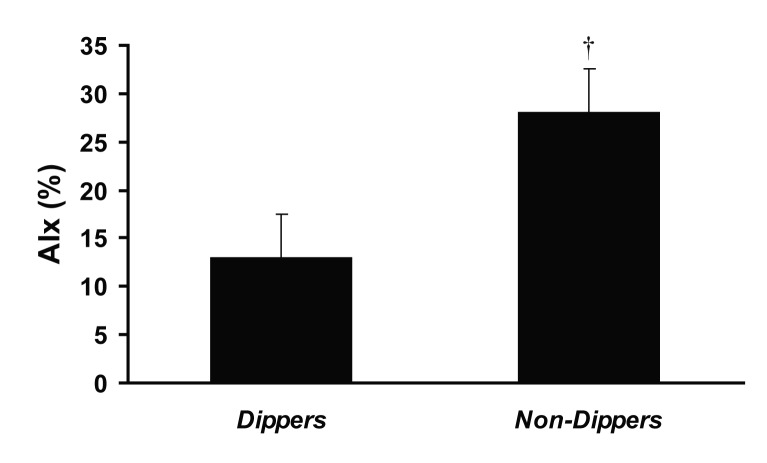
Peripheral arterial tonometery (PAT) derived augmentation index (AIx) in nocturnal dippers versus non-dippers (adjusted for age, BMI, heart rate and mean arterial pressure). ^†^Significantly different from dippers (P < 0.05).

As seen in Figure 2, PAT-AIx was significantly higher in nocturnal non-dippers (n = 13) versus dippers (n = 11) -adjusted for age, gender, mean arterial pressure, body mass index, and heart rate. There were no differences in age, blood pressure, height, heart rate, lipids, medication history, or gender between dippers and non-dippers (Table 1). Moreover, PWA-RHI did not differ between groups (P > 0.05).

## Discussion

PAT-AIx was associated with AASI in patients with hypertension. PAT-AIx was also associated with BPVR and was higher in those patients devoid of nocturnal reduction in BP (i.e. non-dippers) versus those with nocturnal reduction in BP (i.e. dippers). PWA-RHI was not associated with AASI, BPVR and did not differ between dippers and nondippers. Thus PAT-AIx may offer clinical insight into vascular burden and cardiovascular disease risk in patients with hypertension independent of information obtained from PWA-RHI.

The seminal work of Takazawa and colleagues previously used photoplethysmography (PTG) to capture the digital volume waveform [28, 29]. PTG detects the changes in infrared light absorption by hemoglobin in the finger, reflecting pulsatile changes in blood volume. Digital volume traces are very similar to pressure waveforms measured invasively in the ascending aorta [28, 29] and noninvasively in the radial and carotid artery [30]. Moreover changes in the PTG contour via administration of vasoactive agents mirror changes in pressure waveform contour [29, 30]. As such change in AIx derived from the digital volume pulse is highly associated with change in aortic AIx measured from pressure waveforms (r = 0.86, P < 0.05) [28, 29]. Previous studies have noted that the association of AIx derived from PAT and AIx derived from synthesized aortic pressure waves (obtained noninvasively via acquisition of radial artery pressure waveforms and a generalized transfer function) is comparable (r = 0.6-0.9) [31-33].

The ambulatory arterial stiffness index (AASI) is an integrated measure of systemic vascular function derived from 24-hour blood pressure recordings that is associated with target organ damage [15-18] and is an independent predictor of adverse CV events (i.e. stroke and CV mortality) [19-21, 34]. Previous studies have shown that AIx derived from central and peripheral pressure waves were associated with AASI [12]. Li et al reported that AIx accounted for approximately 25% of the variance in AASI [18]. In the present study, PAT-AIx accounted for 27% of the variance in AASI. Thus our results are consistent with previous reports and note similar associations between AIx and AASI, irrespective of the method used to obtain AIx. In the present study, we also noted an association between BPVR and PAT-AIx. The BPVR is another proposed measure of vascular load derived from ambulatory BP monitoring [27]. Recently, Gavish et al demonstrated that this blood pressure independent index was a predictor of short-term mortality in 3433 patients [34]. Our findings suggest that PAT-AIx is associated with these novel measures of CV risk in patients with hypertension.

With sleep, there is a well-noted reduction in blood pressure. Numerous studies note that non-dipping status (attenuated reduction in nocturnal BP) is associated increased arterial stiffness [35, 36], target organ damage such as LV hypertrophy, carotid intima-media thickness [37] and microalbuminuria [38], and higher CV and all-cause mortality [39, 40]. Previous studies have shown that non-dippers have elevated peripheral AIx [41] and our results of elevated PAT-AIx in non-dippers versus dippers is consistent with this finding. Thus, similar to AIx derived from peripheral pressure waveforms, PAT-AIx may provide clinically useful information related to diurnal BP variation patterns and subsequent cardiovascular risk.

PWA-RHI has gained acceptance as a non-invasive measure of microvascular endothelial function. PWA-RHI is associated with conduit and coronary endothelial function [1, 42], cardiovascular risk factors [1, 7, 42] and is predictive of future CV events [2, 3]. In the present study, AIx was not associated with PWA-RHI. Moreover, PWA-RHI was not associated with AASI, BPVR and did not differ between dippers and non-dippers. Thus, both measures obtained from PAT are not mutually inclusive with respect to peripheral vascular appraisal. Each measure appears to provide novel insight into vascular function and cardiovascular risk. In conclusion, PAT-AIx is associated with ambulatory measures of vascular function and may offer clinical insight into vascular burden and cardiovascular disease risk in patients with hypertension independent of information obtained from PWA-RHI.
